# Increased Tenascin C, Osteopontin and HSP90 Levels in Plasmatic Small Extracellular Vesicles of Pediatric ALK-Positive Anaplastic Large Cell Lymphoma: New Prognostic Biomarkers?

**DOI:** 10.3390/diagnostics11020253

**Published:** 2021-02-06

**Authors:** Federica Lovisa, Anna Garbin, Sara Crotti, Piero Di Battista, Ilaria Gallingani, Carlotta Caterina Damanti, Anna Tosato, Elisa Carraro, Marta Pillon, Erfan Mafakheri, Filippo Romanato, Enrico Gaffo, Alessandra Biffi, Stefania Bortoluzzi, Marco Agostini, Lara Mussolin

**Affiliations:** 1 Maternal and Child Health Department, Padova University, 35128 Padova, Italy; federica.lovisa@unipd.it (F.L.); anna.garbin.1@studenti.unipd.it (A.G.); piero.dibattista@phd.unipd.it (P.D.B.); ilaria.gallingani@studenti.unipd.it (I.G.); carlottacaterina.damanti@studenti.unipd.it (C.C.D.); anna.tosato@studenti.unipd.it (A.T.); alessandra.biffi@unipd.it (A.B.); 2 Istituto di Ricerca Pediatrica Città della Speranza, 35127 Padova, Italy; s.crotti@yahoo.it (S.C.); m.agostini@unipd.it (M.A.); 3 Pediatric Hematology, Oncology and Stem Cell Transplant Division, Padova University Hospital, 35128 Padova, Italy; elisa.carraro87@gmail.com (E.C.); marta.pillon@unipd.it (M.P.); 4 Department of Physics and Astronomy, Padova University, 35131 Padova, Italy; emafakheri@gmail.com (E.M.); filippo.romanato@unipd.it (F.R.); 5 IOM-CNR, S.S. 14 km 163,5, 34149 Trieste, Italy; 6 Department of Molecular Medicine, Padova University, 35121 Padova, Italy; enricogaffo@gmail.com (E.G.); stefania.bortoluzzi@unipd.it (S.B.); 7 CRIBI Interdepartmental Research Center for Innovative Biotechnologies (CRIBI), Padova University, 35121 Padova, Italy; 8 First Surgical Clinic Section, Department of Surgical, Oncological and Gastroenterological Sciences, Padova University, 35128 Padova, Italy

**Keywords:** ALCL, small EVs, proteomics, osteopontin, tenascin C, HSP90

## Abstract

Over the past 15 years, several biological and pathological characteristics proved their significance in pediatric anaplastic lymphoma kinase (ALK)-positive anaplastic large-cell lymphoma (ALCL) prognostic stratification. However, the identification of new non-invasive disease biomarkers, relying on the most important disease mechanisms, is still necessary. In recent years, plasmatic circulating small extracellular vesicles (S-EVs) gathered great importance both as stable biomarker carriers and active players in tumorigenesis. In the present work, we performed a comprehensive study on the proteomic composition of plasmatic S-EVs of pediatric ALCL patients compared to healthy donors (HDs). By using a mass spectrometry-based proteomics approach, we identified 50 proteins significantly overrepresented in S-EVs of ALCL patients. Gene Ontology enrichment analysis disclosed cellular components and molecular functions connected with S-EV origin and vesicular trafficking, whereas cell adhesion, glycosaminoglycan metabolic process, extracellular matrix organization, collagen fibril organization and acute phase response were the most enriched biological processes. Of importance, consistently with the presence of nucleophosmin (NPM)-ALK fusion protein in ALCL cells, a topological enrichment analysis based on Reactome- and Kyoto Encyclopedia of Genes and Genomes (KEGG)-derived networks highlighted a dramatic increase in proteins of the phosphatidylinositol 3-kinase (PI3K)/AKT pathway in ALCL S-EVs, which included heat shock protein 90-kDa isoform alpha 1 (HSP90AA1), osteopontin (SPP1/OPN) and tenascin C (TNC). These results were validated by Western blotting analysis on a panel of ALCL and HD cases. Further research is warranted to better define the role of these S-EV proteins as diagnostic and, possibly, prognostic parameters at diagnosis and for ALCL disease monitoring.

## 1. Introduction

Anaplastic large-cell lymphoma (ALCL) accounts for approximately 10–15% of childhood non-Hodgkin lymphomas [1]. In the vast majority of cases, ALCL is characterized by constitutive expression of anaplastic lymphoma kinase (ALK) fusion proteins due to the presence of chromosomal translocations involving the ALK gene on chromosome 2, the most frequent of which is the t(2;5)(p23;q35), juxtaposing the *ALK* kinase domain coding sequence to *NPM1* oligomerization domain [2].

In recent years, several biological features emerged as promising prognostic factors for the clinical management of pediatric ALCL, including non-common histology, minimal disseminated and residual disease and anti-ALK antibody titers [3,4]. Despite a continuous improvement in the field of ALCL prognostic stratification, the identification of new, non-invasive disease biomarkers which might further complement those currently in use, while relying on the most relevant disease mechanisms, is still necessary. In this regard, extracellular vesicles (EVs) gathered great importance both as stable biomarkers carriers and as active players in tumorigenesis. EVs’ nomenclature and classification have been recently updated by the International Society for Extracellular Vesicles (ISEV), which classifies EVs as large (L)- and small (S)-EVs according to their diameter. L-EVs generally include microvesicles or ectosomes (>500 nm), directly budding from the plasma membrane, apoptotic bodies (0.8–5 µm) and large oncosomes (1–10 µm), whereas S-EVs mainly include exosomes (<150/200 nm) and non-membranous exomeres (~35 nm) [5]. While plasma membrane-budded microvesicles are predominantly characterized as products of platelets, endothelial cells and red blood cells, S-EVs are intraluminal vesicles deriving from multivesicular bodies formed inside endosomal compartments [6]. During this process, several mechanisms ensure the specific sorting of bioactive molecules into exosomes, making them the most attractive intercellular messengers among all EVs [7,8,9]. The nucleic acids, lipids and the protein cargo in these S-EVs are involved in several oncogenic processes, including cell proliferation, angiogenesis, immune system modulation and metastasis [10]. In particular, S-EV proteins’ ability to exert direct functions in distant sites and to target specific organs makes them promising tumor-specific biomarkers [11].

Mass spectrometry (MS)-based proteomics has emerged as a broadly effective means for identifying, characterizing and quantifying proteins in biological samples. Proteomic analyses of plasmatic S-EVs offer an attractive avenue to increase our understanding of the biological processes involved in disease onset and dissemination and to translate results from the laboratory into the clinic, with the identification of potential disease biomarkers [12].

To date, data on the proteomic composition of plasmatic S-EVs in ALCL patients are still missing. In the present work, we performed extensive research on the proteomic make-up of plasmatic S-EVs of ALCL patients compared to healthy donors, and we propose S-EV tenascin C (TNC), osteopontin (SPP1/OPN) and heat shock protein 90-kDa isoform alpha 1 (HSP90AA1) as potential biomarkers for ALCL prognostic stratification.

## 2. Materials and Methods

### 2.1. Patient Samples

Plasma samples were collected in our laboratory, which is the national reference laboratory for the centralized molecular diagnosis of pediatric non-Hodgkin lymphomas enrolled in the “Associazione Italiana di Ematologia e Oncologia Pediatrica” (AIEOP) treatment protocols. All ALCL cases were diagnosed by immunohistochemistry using a wide panel of antibodies recognizing T-lineage and B-lineage markers (CD2, CD3, CD4, CD5, CD7, CD8, CD20, CD43, CD45RO, CD79a), Natural Killer (NK) markers (CD56, CD57), Alk-1, epithelial membrane antigen (EMA) and CD30. Histological and immunohistochemical diagnoses were centrally reviewed. All patients were treated according to the international ALCL-99 protocol [13].

Peripheral blood samples in sodium citrate (5 mL) were collected before treatment initiation and processed in the laboratory within 24 h. Briefly, the samples were centrifuged at 820× *g* for 10 min and supernatants were carefully removed and then re-centrifuged at 2500× *g* for 10 min to avoid contamination by platelets. Plasma aliquots were stored in the Pediatric Oncology Biobank (BBOP) at −80 °C until used. The samples included in the proteomic study were from 20 ALCL patients and five healthy donors (HDs). Of these, four ALCL patients from the same cohort, five other ALCL patients and three other HDs were used to validate mass spectrometry data by Western blotting. The main clinical and biological characteristics of the study cohort are reported in Table 1 and Table S1 (Supplementary Materials). The study was approved by the local ethics committee and informed consent was obtained from parents or legal guardians before patient enrollment.

### 2.2. S-EV Enrichment and Quantitation

Plasma samples (500 µL) were filtered to exclude particles larger than 0.22 µm and processed using the exoEasy midi kit (Qiagen, Hilden, Germany), following the manufacturer’s instructions. Enriched S-EVs were eluted in 300 µL XE Buffer and quantified using the FluoroCet Exosome Quantitation Kit (System Biosciences, Palo Alto, CA, USA), following the manufacturer’s protocol.

### 2.3. Transmission Electron Microscopy (TEM)

TEM analysis to confirm plasma S-EVs’ morphology was performed on pellets of purified S-EVs loaded on formvar/carbon-coated grids. Furthermore, 2% Ammonium-Molybdate was used as a standard negative stain in biological electron microscopy before mounting in the sample position of the microscope. S-EVs were appropriately diluted to form a thin layer on the EM grid in order to afford the transmission of the electron beam. A Tecnai G2 Spirit TEM (FEI Company, Hillsboro, OR, USA) was used to image S-EV samples with a diameter ranging between 30 and 150 nm and magnified up to 300 kX.

### 2.4. Nanoparticle Tracking Analysis

Nanoparticle tracking analysis (NTA) was conducted on a Nanosight NS300 instrument (Malvern Panalytical, Malvern, UK), which allows particles’ visualization and analysis in liquids, relating the rate of Brownian motion to particle size. The instrument was equipped with a 488-nm laser, a high-sensitivity sCMOS camera and a syringe pump. The plasmatic S-EV samples were mixed by vortexing and subsequently diluted at 1:1000 in particle-free PBS 1X to obtain a concentration within the recommended measurement range (1–10 × 10^8^ particles/mL). Experiment videos were analyzed using NTA 3.1 build 54 software (Malvern Panalytical) after capture in script SOP Standard Measurement (3 videos of 60 s per measurement), using a syringe pump speed of 30. A total of 1500 frames were examined per sample.

### 2.5. Proteomics,

S-EVs from 20 ALCL patients and 5 HDs were sonicated in an ultrasonic water bath for 10 min in ice. Lysates were quantified using the Bicinchoninic Acid (BCA) Protein assay kit (ThermoFisher Scientific, Waltham, MA, USA) and 15 μg of protein per sample was diluted in 50 mM ammonium bicarbonate (pH ~8). Samples were then reduced by adding 100 mM dithiothreitol (DTT) at 60 °C for 45 min and alkylated by adding 100 mM iodoacetamide (IAA) at room temperature for 30 min, in the dark. Overnight digestion was performed by adding MS sequencing-grade trypsin (Promega, Madison, WI, USA) in a 1:25 (trypsin: protein) ratio. The reaction was stopped by adding a 10% trifluoroacetic acid (TFA) solution. Digested proteins were purified through C18 SPE cartridges (Supelco, Bellefonte, PA, USA) and finally dried under vacuum until analysis. Peptides were resuspended in 25 μL of 5% CH_3_CN + 0.025% TFA before injection. An Ultimate 3000 HPLC system coupled to a Q Exactive (ThermoFisher Scientific) mass spectrometer was employed. The peptide mixtures were injected onto a Biobasic C18 column, 5-μm particle size, 1 × 15 mm, and separated using a 3–45% linear gradient of CH_3_CN + 0.025% TFA (both supra-pure grade, Romil, Cambridge, UK) in H_2_O + 0.025% TFA at 110 min of analysis. Mass spectrometry data were acquired for each individual sample in data-dependent mode in the 300–1500 m/z mass range. Instrumental parameters were set as follows: source: ESI (+); precursor charge selection: from 2 to 5; precursor resolution: 17,500; fragments resolution: 60,000.

LC-MS/MS data were processed by Proteome Discoverer 2.2 (ThermoFisher Scientific) using the Sequest HT algorithm for protein identification. Search parameters were set as follows: database, SwissProt (25 October 2017); enzyme, Trypsin (max 2 missed cleavages); taxonomy, *Homo sapiens*; precursor mass tolerance, 10 ppm; fragment mass tolerance, 0.02 Da. Fixed modification: carbamidomethyl (C). Dynamic modifications: oxidation (M, P), deamidation (N, Q) and phosphorylation (S, T, Y).

### 2.6. Bioinformatic Analysis and Network Reconstruction

Obtained protein abundance data in arbitrary units (AU) were filtered by discarding known plasmatic proteins. Statistical analyses were carried out using in-house scripts in R statistical software (v3.4.4). Significant differences in protein abundance between ALCL and HD S-EVs were detected using the Wilcoxon rank sum test, and the Benjamini–Hochberg adjustment was applied to control the false discovery rate (FDR). Proteins with adjusted *p*-value ≤ 0.1 were considered to have a significantly different abundance between considered groups.

DAVID v6.8 [14,15] was used to perform Gene Ontology enrichment analysis. Cytoscape v3.8.1 was used to build, annotate and visualize the protein network obtained using topological enrichment analysis, based on Reactome and Kyoto Encyclopedia of Genes and Genomes (KEGG) pathway-derived networks, provided by the Reactome FI PlugIn of Cytoscape.

### 2.7. Western Blotting

S-EVs in XE buffer were lysed in 2X RIPA buffer (150 mM NaCl, 1% Triton X-100, 0.1% SDS, 1% sodium deoxycholate, 25 mM Tris-HCL, pH 8.0, 1:100 phosphatase inhibitor cocktails 2 and 3, Sigma-Aldrich, St. Louis, MO, USA) for 5 min at 37 °C and centrifuged at 14,000× *g* for 5 min. Protein amounts were determined using the Pierce Micro BCA Protein Assay kit (ThermoFisher Scientific). Proteins from peripheral blood mononuclear cells (PB-MNC) were isolated using Trizol Reagent (ThermoFisher Scientific) following the manufacturer′s instructions. Protein concentrations were measured using the BCA protein assay (ThermoFisher Scientific).

For Western blotting analysis, 20 μg of S-EVs lysates and 30 μg of PB-MNC lysates were resolved by SDS-PAGE, prior to being transferred onto a nitrocellulose membrane (PerkinElmer, Waltham, MA, USA). Blocked membranes were probed for HSP90alpha (rat, 1:1000, Enzo Life Sciences, Farmingdale, NY, USA), Calnexin (CANX) (mouse, 1:1000, Santa Cruz Biotechnology, Dallas, TX, USA), CD9 (rabbit, 1:1000, System Biosciences), SPP1/OPN (mouse, 1:1000, Santa Cruz Biotechnology) and TNC (rabbit, 1:1000, Abcam, Cambridge, UK). Membranes were digitally acquired by using an iBright FL1500 imaging system (ThermoFisher Scientific). Band densities were analyzed using ImageJ software [16] and normalized on CD9 expression as a loading control. The expression levels of TNC, SPP1 and HSP90alpha between HD and ALCL were compared using the Mann–Whitney test.

## 3. Results

### 3.1. Evaluation of S-EV Markers and Vescicle Size and S-EV Quantitation

**Figure 1:**
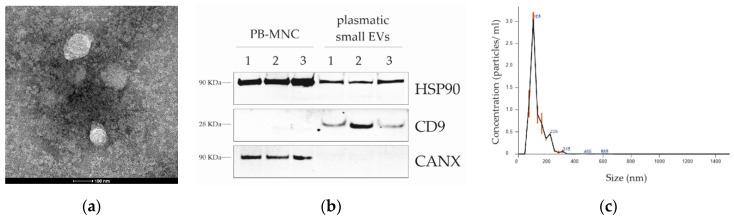
ALCL plasmatic small extracellular vesicle (S-EV) characterization. (**a**) Transmission electron microscopy analysis of plasmatic S-EVs from a representative ALCL sample. (**b**) Western blotting analysis of peripheral blood mononuclear cells (PB-MNCs) and representative ALCL plasmatic S-EVs, showing the presence of heat shock protein 90-kDa (HSP90) protein in both whole cells and S-EV lysates, exosome-specific tetraspanin CD9 in S-EV lysates only and the endoplasmic reticulum marker Calnexin (CANX) in total lysates, but not in S-EVs. (**c**) Size distribution of particle diameters (nm) of vesicles isolated from a representative ALCL plasma sample, as measured by nanoparticle tracking analysis (Nanosight, Malvern Panalytical).

To confirm the enrichment of S-EVs from plasma-processed samples, vesicle suspensions in XE buffer were subjected to S-EV marker and size analyses (Figure 1). S-EV lysates, resolved by SDS-PAGE, showed the exosomal-specific markers CD9 [17] and HSP90alpha [18] and were negative for Calnexin (CANX), an endoplasmic reticulum protein (Figure 1a) that was present, instead, in PB-MNC total lysate. Size analysis, conducted by TEM and NTA, showed that S-EV samples contained particles mainly within 50 and 150 nm (Figure 1b,c) [19] and supporting the samples’ enrichment in S-EVs. S-EV quantitation using the FluoroCet Exosome Quantitation Kit confirmed that comparable S-EV amounts were obtained from the same starting plasma volume (500 µL) of 5 HDs and 14 ALCL cases (Figure S1).

### 3.2. Proteomic Profile of ALCL Plasmatic S-EVs

To determine the protein composition of ALCL S-EVs in comparison to HD subjects, a proteomic analysis was performed as described in the experimental section. Overall, 1798 proteins were identified, 1021 of which were detected with at least two unique peptides. After removal of the overlapping protein isoforms, a total of 277 non-redundant bona fide S-EV proteins with median abundance above the first quartile were obtained, for which the absolute intensity, as estimated by label-free quantification, was calculated. The specific S-EV marker CD9 was detected in S-EVs from both ALCL patients and HDs (Figure S2).

**Figure 2:**
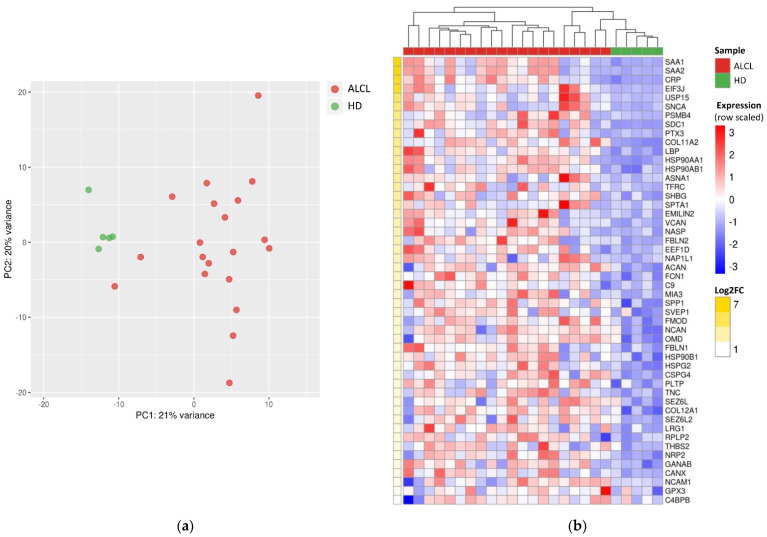
Differentially abundant proteins in S-EVs from ALCL samples and healthy donors. (**a**) Principal component analysis performed on abundance levels of 277 identified proteins distinguishes ALCL from healthy donor (HD) S-EVs. (**b**) Heatmap of the 50 proteins overrepresented in ALCL S-EVs, ordered by decreasing log_2_fold change (LFC). Unsupervised hierarchical clustering revealed that ALCL S-EV samples have a unique protein expression profile, distinct from HD.

Principal component analysis of S-EVs’ protein abundance profiles showed a clear separation of ALCL and HD samples (Figure 2a). Statistical analysis of quantification data identified 83 proteins with significantly different abundance in ALCL cases compared to HDs, 50 of which were overrepresented and 33 which were less represented in S-EVs of patients (Table S2).

To investigate those proteins possibly playing an active role in the disease, we focused on the 50 polypeptides overrepresented in ALCLs (Figure 2b). Serum amyloid A isoforms SAA1 and SAA2 and C-reactive protein (CRP) were dramatically overrepresented in ALCLs (log_2_fold change > 6; Table S2). Intriguingly, these proteins’ highest abundance was observed in two patients that subsequently experienced a disease relapse (Figure S3a–c). Another acute phase protein, lipopolysaccharide binding protein (LBP), was significantly abundant in ALCL S-EVs (log_2_fold change = 3.48; Table S2), with the highest levels in two patients that later relapsed (Figure S3d).

### 3.3. Proteins of the PI3K/AKT Pathway Modules are Enriched in ALCL Exosomes

**Figure 3:**
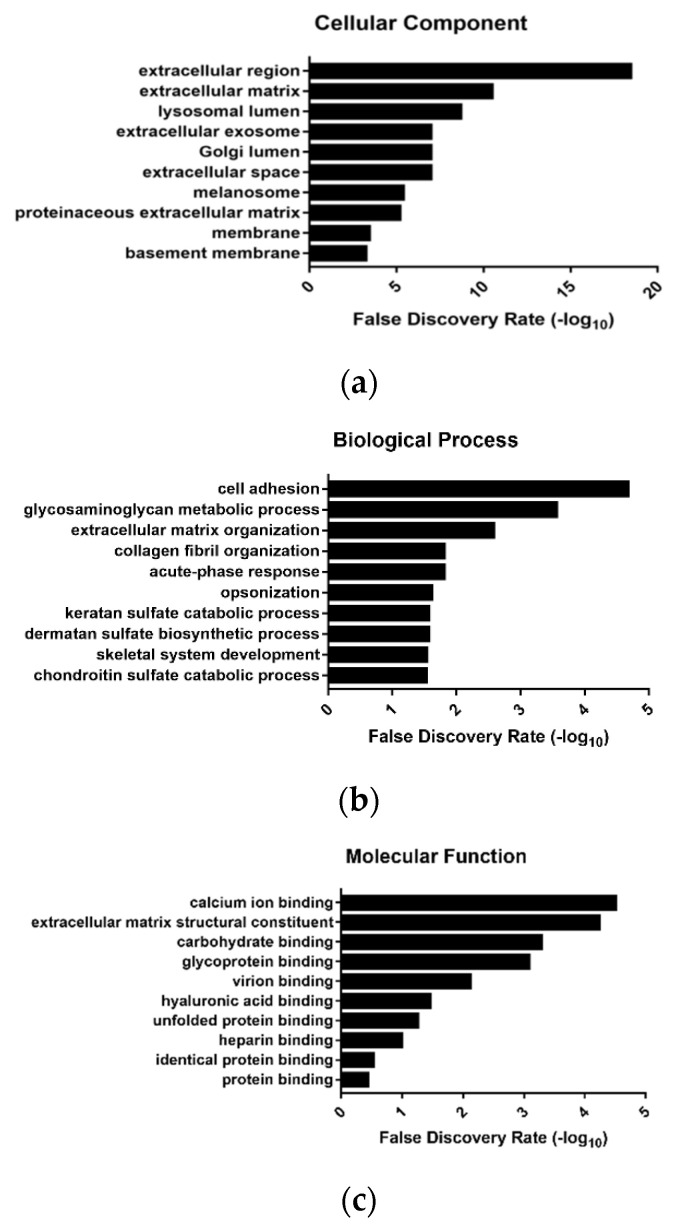
Gene Ontology (GO) distribution of proteins more abundant in ALCL samples. The top 10 significantly enriched GO terms in ALCL S-EVs: cellular component (**a**), biological process (**b**) and molecular function (**c**). Only GO terms represented by ≥3 genes were considered and ranked according to decreasing −log_10_ of false discovery rate (FDR).

Further investigation focused on the possible functional roles of the protein cargos enriched in ALCL S-EVs. In agreement with exosome biogenesis, the cellular component ontologies most enriched in the 50 proteins overrepresented in ALCLs belonged to extracellular region, extracellular matrix, lysosomal lumen, extracellular exosome and Golgi lumen (Figure 3a; Table S3). Several proteins carried in ALCL S-EVs were implicated in cell adhesion, glycosaminoglycan metabolic process, extracellular matrix organization, collagen fibril organization and acute phase response. The most enriched molecular functions were related to vesicular trafficking processes: calcium ion binding, extracellular matrix structural constituent, carbohydrate binding and glycoprotein binding (Figure 3b,c; Tables S4 and S5).

**Figure 4:**
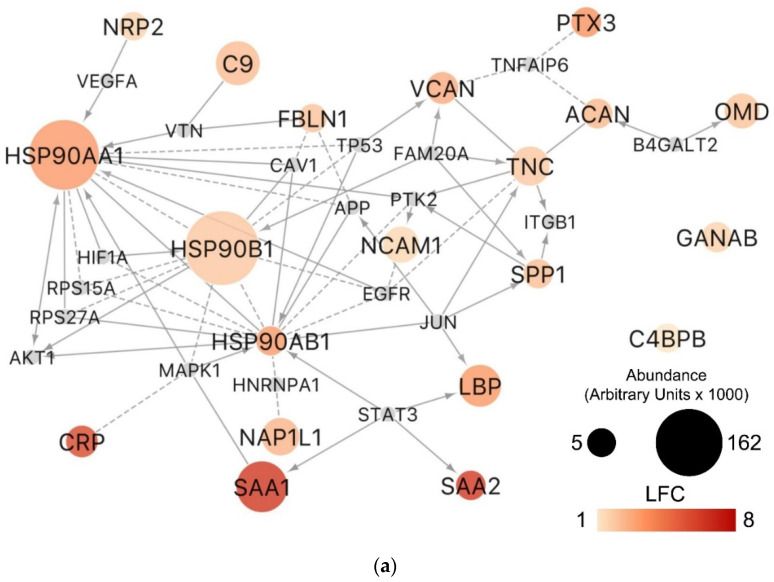

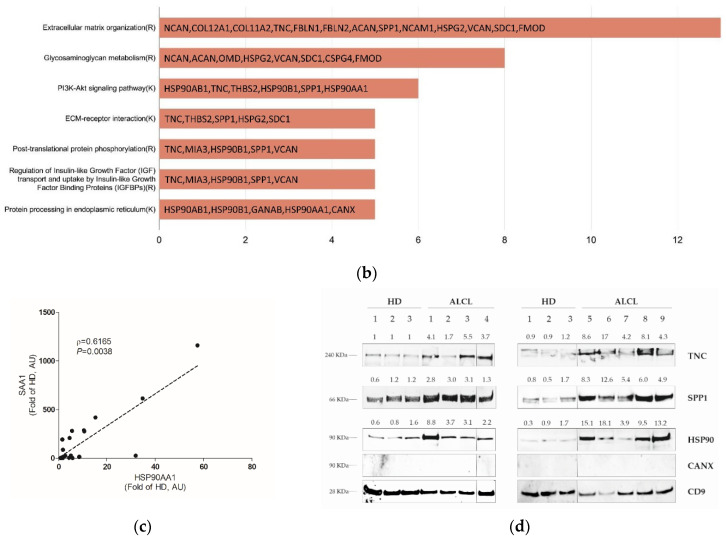
Pathway modules involving proteins overrepresented in S-EVs of ALCL patients highlight phosphatidylinositol 3-kinase (PI3K)/AKT signaling pathway proteins in the plasma S-EVs cargo in ALCL. (**a**) Pathway-derived network and (**b**) Reactome and Kyoto Encyclopedia of Genes and Genomes (KEGG) modules significantly enriched (FDR < 0.01) by the proteins overrepresented in ALCL S-EVs. In the network, node size indicates the protein abundance (arbitrary units ×1000) in ALCL; color shades indicate the log_2_ fold change (LFC) of protein abundance with respect to healthy donors (HD). Only the overexpressed proteins with average abundance ≥ 5000 in ALCL are shown. Small grey nodes represent first neighbors connecting two or more of the proteins overrepresented in ALCL S-EVs. Edges indicate different types of experiments from which the relationship was determined: expression regulation (arrow), complex (solid line) or predicted (dashed line). (**c**) Scatterplot showing the Spearman correlation between heat shock protein 90 isoform alpha 1 (HSP90AA1) and serum amyloid A1 (SAA1) in 20 ALCL-derived S-EVs (*ρ* = 0.6165, *p* = 0.0038). Data in arbitrary units (AU) are expressed as fold of HD. (**d**) Western blotting of tenascin C (TNC), osteopontin (SPP1) and HSP90 in ALCL and HD samples. Band densities were acquired using ImageJ software and expressed as a fold of HD bands’ mean density. CD9 was used as a loading control. Vertical lines were inserted to indicate repositioned gel lanes. Expression levels between ALCLs and HDs were compared by using the Mann–Whitney test.

Furthermore, topological enrichment analysis based on Reactome- and KEGG-derived networks identified 20 out of the 50 proteins enriched in ALCL S-EVs that are connected to each other directly or by a first-degree neighbor (Figure 4a). We identified four Reactome and three KEGG pathway modules significantly overrepresented among these functionally connected proteins (Figure 4b). Several proteins were involved in the extracellular matrix organization, including TNC, Fibulin 1 (FBLN1), Aggrecan (ACAN), SPP1, Neural Cell Adhesion Molecule 1 (NCAM1) and Versican (VCAN) among the most abundant S-EV polypeptides. Proteins of the phosphatidylinositol 3-kinase (PI3K)/AKT signaling pathway were of particular interest since the nucleophosmin (NPM)-ALK fusion protein of ALCL cells is known to activate several interconnected oncogenic signaling pathways, including RAS-extracellular signal-regulated kinase (ERK), Janus kinase 3 (JAK3)- Signal Transducer and Activator of Transcription 3 (STAT3) and phosphatidylinositol 3-kinase (PI3K)-AKT [20]. HSP90AA1, HSP90AB1, HSP90B1, SPP1, Thrombospondin 2 (THBS2) and TNC proteins belonging to the PI3K/AKT pathway presented a dramatic increase in ALCL S-EVs (1.5 to 3.5 (LFC)). HSP90AA1 and HSP90AB1 were particularly abundant (Figure 4a; Table S2) and the highest levels were detected in three and two patients who subsequently relapsed, respectively (Figure S3d,e). Moreover, the SAA1 protein, having a particularly marked increase in ALCL samples, is directly linked to STAT3 (Figure 4a), which is a known downstream target of the NPM-ALK fusion protein. A significant correlation between HSP90AA1 and SAA1 circulating levels was also observed (Figure 4c). By Western blotting analysis, we confirmed that TNC, SPP1 (better known as OPN) and HSP90AA1 were significantly more abundant in ALCL plasmatic S-EVs compared to HD (Figure 4d, Mann–Whitney *p* = 0.0004, *p* = 0.0008 and *p* = 0.0004, respectively).

## 4. Discussion

In this study, we performed a comprehensive characterization of proteins circulating in ALCL patients’ plasma S-EVs and we provide preliminary observations on new potential biomarkers for ALCL prognostic stratification.

Among the most abundant proteins found in ALCL S-EVs, we identified serum amyloid A (SAA) protein isoforms SAA1 and SAA2. SAA acute phase proteins are mainly produced by the liver under the regulation of inflammation-associated cytokines during acute and chronic inflammatory processes [21]. However, SAA can also be synthesized in extrahepatic tissues, including primary and metastatic cancer cell lines [22]. In recent years, a close connection between malignant transformation and inflammation has been discovered in several clinical contexts, and SAA has been proposed as a marker of tumor progression in solid tumors [23]. In hematological malignancies, including both Hodgkin and non-Hodgkin lymphomas, SAA levels were generally elevated and correlated with advanced diseases [24]. In line with this, although in our samples most of the patients (18 out of 20) presented with disease stage III/IV, the lowest levels of S-EV SAA1 and SAA2 were detected in two patients with stage I/II, with the stage I patient showing undetectable levels of SAA2 (Table S1). Of interest, higher levels of SAA1 and SAA2 proteins were detected in S-EVs from patients that subsequently relapsed, though not reaching statistical significance (Figure S3a,b).

Another acute phase protein, namely the lipopolysaccharide binding protein (LBP), was significantly increased in ALCL S-EVs, with particularly higher levels in two cases that subsequently relapsed (Figure S1d). Interestingly, LBP was found to be abundant in circulating exosomes of non-small cell lung cancer patients with metastatic disease [25], thus suggesting that increased circulating LBP might be associated with a more aggressive disease.

Our results also indicated that proteins of the PI3K/AKT pathway circulate in ALCL patients’ bloodstream within S-EVs. Particularly, HSP90AA1 is the human stress-inducible 90-kDa heat shock protein isoform alpha 1, which is the major form of HSP90 protein, whereas HSP90AB1 is the constitutive expressed and minor form. HSP90 is the core component of a chaperone machinery involved in controlling the function and activity of several hundreds of protein substrates. Its importance in maintaining NPM-ALK structural integrity and function has been also shown in ALK-positive ALCL cell lines [26]. Our findings of a significant increase in both HSP90AA1 and HSP90AB1 could be explained both by the presence of elevated levels of the NPM-ALK chimeric protein and/or by a diffused inflammatory state, as suggested by the elevated levels of SAA1, SAA2 and CRP proteins [27]. Indeed, HSP90AA1 was directly linked to SAA1 downstream of STAT3 activity, and a significant correlation between HSP90AA1 and SAA1 levels was observed in our proteomics data. Overall, these findings suggest that both inflammation and NPM-ALK signaling might participate in inducing high HSP90 circulating levels and in defining the composition of ALCL S-EV proteomes. Furthermore, the link between HSP90 and HIF1A, a regulator of hypoxia-induced angiogenesis [28], elicits the speculation that HSP90 delivery by S-EVs could play a role in supporting tumor growth and promoting metastasis formation. Despite the small number of relapsed patients available, both HSP90AA1 and HSP90AB1 seem to be increased in patients that subsequently relapsed (Figure S1e,f), pending confirmation on a larger patient cohort.

Osteopontin (SPP1/OPN) is an acidic arginine-glycine-aspartate containing adhesive glycoprotein, initially discovered in transformed, malignant epithelial cells [29,30]. Dysregulation of SPP1 is linked to many diseases including autoimmune disorders, inflammatory diseases and fibrosis. Several studies found increased SPP1 levels in different types of cancer [31,32,33,34]. SPP1 sustains angiogenesis via PI3K/AKT- and ERK-mediated pathway activation [35] and by interacting with αVβ3 integrin to feed endothelial cells’ survival [30]. Moreover, SPP1 plays a crucial role in epithelial–mesenchymal transition [30], and high SPP1 expression levels have also been associated with metastasis formation in colorectal cancers, lung cancers and melanomas [36]. Our study demonstrates, for the first time, that abnormally high levels of SPP1 are present in S-EVs circulating in the blood of pediatric ALCL patients, with higher levels in two patients that subsequently relapsed (Figure S3g). In a recent study, increased SPP1 expression was detected in diffuse large B-cell lymphoma by tissue microarray analysis, particularly in patients with reduced overall survival [37]. Similar to our findings in pediatric ALCL patients, circulating SPP1 was observed to be significantly higher in adult non-Hodgkin lymphoma patients compared to HDs [38].

Tenascin C (TNC) is a large hexameric glycoprotein expressed in the extracellular matrix of the connective tissue [39]. *TNC* is also expressed in malignant tissues, particularly upon initiation of angiogenesis [40], and its expression, both in cancer-associated stroma and tumor cells, has been associated with a poor clinical outcome [41,42]. In ALCL tumor tissues, TNC is strongly and diffusely expressed in stromal cells, vasculature and tumor cells [43]. We highlighted the presence of more than twice the amount of TNC in plasmatic S-EVs of ALCL patients with respect to HD samples. Since a significant association has been previously found between the proportion of lymph node-involved areas and prognosis in adult T-cell non-Hodgkin lymphomas [43], S-EV-associated TNC might represent a promising biomarker for ALCL, and further research is warranted to define whether higher TNC levels may be characteristic of a more aggressive disease (Figure S3h).

The small number of patients included in the analysis and the short follow-up time for most of them represent the major limitations of this study. A more quantitative and rapid method, such as specific ELISA assays, has to be applied, hopefully, to screen a large cohort of ALCL patients, both at diagnosis and during follow-up, to evaluate the correlation between biomarker expression and the risk of relapse.

Nevertheless, our proteomic analysis of pediatric ALCL plasmatic S-EVs suggests TNC, SPP1 and HSP90 as potential prognostic biomarkers for pediatric ALCL disease. In addition, the acute phase proteins SAA1, SAA2, CRP and LBP showed increased levels in ALCL patients compared to HDs, with particularly high levels in some cases that subsequently relapsed. Overall, our findings pave the way for further investigation of these proteins in ALCL disease progression.

## Figures and Tables

**Table 1:** Clinical characteristics at diagnosis of the anaplastic large-cell lymphoma (ALCL) study cohort.

Characteristic	No. of Patients (N = 25)
Gender	
Male	9 (36%)
Female	16 (64%)
Age, years	
<10	12 (48%)
≥10	13 (52%)
Stage at diagnosis	
I-II	4 (16%)
III-IV	21 (84%)
Histology	
Common type	13 (52%)
Non-common type	6 (24%)
Unknown	6 (24%)
Minimal Disseminated Disease	
Positive	16 (64%)
Negative	9 (36%)

## Data Availability

All data are included in the paper.

## Supplementary Materials

The following are available online at https://www.mdpi.com/2075-4418/11/2/253/s1, Table S1: Clinical and biological characteristics of ALCL patients; Table S2: List of proteins with different relative abundance in ALCL cases compared to healthy donors; Table S3: Cellular components distribution of upregulated proteins; Table S4: Biological processes distribution of upregulated proteins; Table S5: Molecular function distribution of upregulated proteins; Figure S1: Small EVs quantitation in ALCL and HD; Figure S2: CD9 expression in S-EV from ALCL patients and HD; Figure S3: Expression levels of candidate ALCL biomarkers as measured by LC-MS/MS.
