# Supplementary material for: Increased Tenascin C, Osteopontin and HSP90 Levels in Plasmatic Small Extracellular Vesicles of Pediatric ALK-Positive Anaplastic Large Cell Lymphoma: New Prognostic Biomarkers?
Source: Diagnostics (Basel). 2021 Feb 6;11(2):253. doi: 10.3390/diagnostics11020253 (PMC7915848; doi:10.3390/diagnostics11020253)
Supplement: Supplementary file 1 [file diagnostics-11-00253-s001.zip › Supplementary material.pdf]

Increased tenascin C, osteopontin and HSP90 levels in plasmatic small extracellular vesicles of pediatric ALK-positive anaplastic large cell lymphoma: new prognostic biomarkers?

Federica Lovisa, Anna Garbin, Sara Crotti, Piero Di Battista, Ilaria Gallingani, Carlotta C. Damanti, Anna Tosato, Elisa Carraro, Marta Pillon, Erfan Mafakheri, Filippo Romanato, Enrico Gaffo, Alessandra Biffi, Stefania Bortoluzzi, Marco Agostini and Lara Mussolin

Supplementary Material

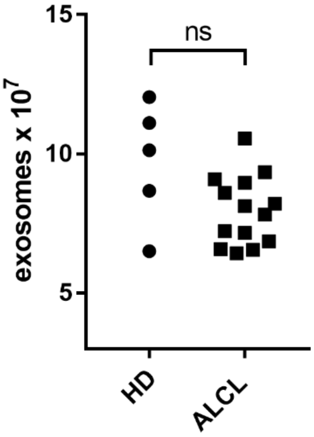

Figure S1. Small EVs quantitation in ALCL and HD. Dotplots representing the number of small-EVs (S-EVs) obtained from 500 µl plasma of 5 HDs and 14 ALCLs. S-EVs were obtained by using the exoEasy midi kit (Qiagen). Quantification has been performed by the Fluorocet Exosome Quantitation Kit (System Biosciences).

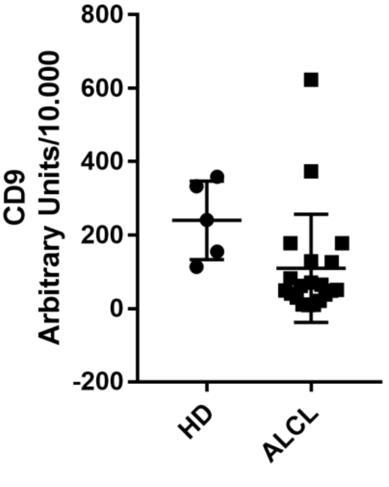

Figure S2. CD9 expression in S-EVs from ALCL patients and HDs. Dotplots of CD9 expression as measured by LC-MS/MS. Data are expressed in arbitrary units/10.000.

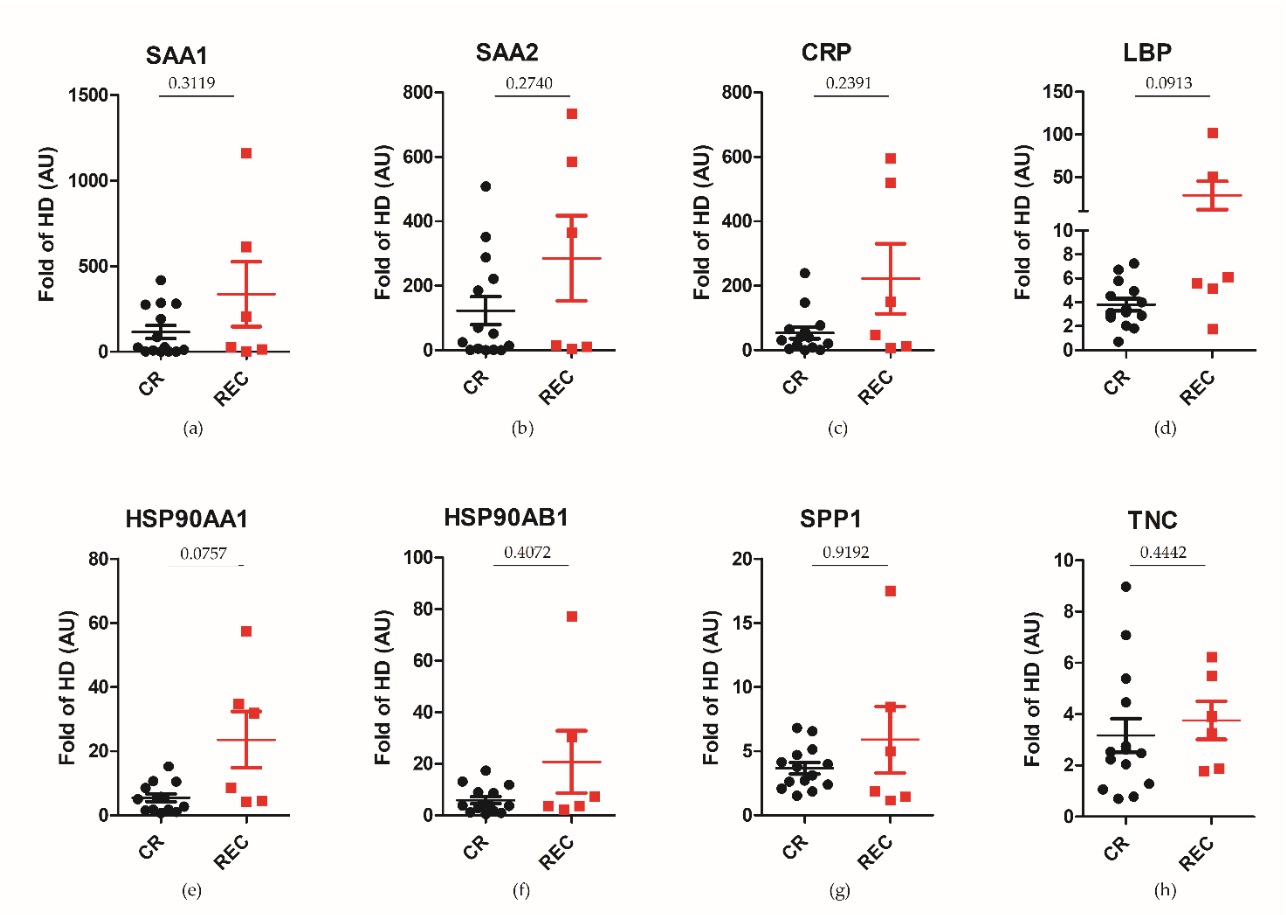

Figure S3. Expression levels of candidate ALCL biomarkers as measured by LC-MS/MS. Dotplots representing the expression of SAA1, SAA2, CRP, LBP, HSP90AA1, HSP90AB1, SPP1 and TNC in non-relapsed (CR) and relapsed (REC) ALCL patients at diagnosis. Data in Arbitrary Units (AU) are expressed as fold of healthy donors (HD). Mann-Whitney test was applied to compare the expression between CR and REC cases.
